# The GP-45 Protein, a Highly Variable Antigen from *Babesia bigemina*, Contains Conserved B-Cell Epitopes in Geographically Distant Isolates

**DOI:** 10.3390/pathogens11050591

**Published:** 2022-05-18

**Authors:** Miguel Angel Mercado-Uriostegui, Luis Alberto Castro-Sánchez, Gaber El-Saber Batiha, Uriel Mauricio Valdez-Espinoza, Alfonso Falcón-Neri, Juan Alberto Ramos-Aragon, Ruben Hernández-Ortiz, Shin-Ichiro Kawazu, Ikuo Igarashi, Juan Mosqueda

**Affiliations:** 1 Immunology and Vaccines Laboratory, College of Natural Sciences, Autonomous University of Queretaro, Santiago de Querétaro 76140, Querétaro, Mexico; miguel.mercado.uriostegui@hotmail.com (M.A.M.-U.); mvzluiscastro@gmail.com (L.A.C.-S.); umvaldez@gmail.com (U.M.V.-E.); 2 Programa de Doctorado en Ciencias biológicas, Facultad de Ciencias Naturales, Universidad Autónoma de Querétaro, Juriquilla 76230, Querétaro, Mexico; 3 National Research Center for Protozoan Diseases, Obihiro University of Agriculture and Veterinary Medicine, Hokkaido 080-8555, Japan; gaberbatiha@gmail.com (G.E.-S.B.); skawazu@obihiro.ac.jp (S.-I.K.); igarcpmi@obihiro.ac.jp (I.I.); 4 Department of Pharmacology and Therapeutics, Faculty of Veterinary Medicine, Damanhour University, Damanhour 22511, AlBeheira, Egypt; 5 Facultad de Medicina Veterinaria y Zootecnia, Universidad Nacional Autonoma de Mexico, Coyoacán 04510, Ciudad de Mexico, Mexico; 6 CENID-Salud Animal e Inocuidad/INIFAP, Carretera Federal Cuernavaca-Cuautla #8534, Col. Progreso, Jiutepec 62550, Morelos, Mexico; alfalconeri@hotmail.com (A.F.-N.); ros1958k@hotmail.com (J.A.R.-A.); hernandez.ruben@inifap.gob.mx (R.H.-O.)

**Keywords:** *B. bigemina*, indirect ELISA, B-cell epitopes, GP-45

## Abstract

In *B. bigemina*, the 45 kilodaltons glycoprotein (GP-45) is the most studied. GP-45 is exposed on the surface of the *B. bigemina* merozoite, it is believed to play a role in the invasion of erythrocytes, and it is characterized by a high genetic and antigenic polymorphism. The objective of this study was to determine if GP-45 contains conserved B-cell epitopes, and if they would induce neutralizing antibodies. The comparative analysis of nucleotide and amino acids sequences revealed a high percentage of similarity between field isolates. Antibodies against peptides containing conserved B-cell epitopes of GP-45 were generated. Antibodies present in the sera of mice immunized with GP-45 peptides specifically recognize *B. bigemina* by the IFAT. More than 95% of cattle naturally infected with *B. bigemina* contained antibodies against conserved GP-45 peptides tested by ELISA. Finally, sera from rabbits immunized with GP-45 peptides were evaluated in vitro neutralization tests and it was shown that they reduced the percentage of parasitemia compared to sera from rabbits immunized with adjuvant. GP-45 from geographically distant isolates of *B. bigemina* contains conserved B-cell epitopes that induce neutralizing antibodies suggesting that this gene and its product play a critical role in the survival of the parasite under field conditions.

## 1. Introduction

Bovine babesiosis is a parasitic disease caused by the intraerythrocytic protozoa of the genus *Babesia* [1]. In the Americas, it is transmitted mainly by *Rhipicephalus microplus* and *R. annulatus* ticks [2]. In addition to *Babesia* species causing economic losses and deaths in cattle, there are also species that can parasitize humans, small ruminants [3], domestic animals and wildlife [4]. Bovine babesiosis caused by *Babesia bigemina* and *B. bovis* has a high impact on the livestock industry in the Americas, affecting the production of meat, milk and leather [5]. It is estimated that around 2 billion cattle around the world are exposed to babesiosis [3]. Ghosh et al. [6] estimated that tick-borne diseases and their treatments cause economic losses ranging between USD 13.9 and 18.7 billion per year worldwide. 

*Babesia* merozoites bind to erythrocytes ligands with their membrane surface proteins. There are several well-characterized membrane proteins that serve as anchors for the parasite to adhere to receptors on the surface of the erythrocyte. However, some of these proteins are highly variable because *Babesia* merozoites experience a high degree of variability in their membrane proteins to evade the immune system [7]. One of these membrane proteins is GP-45, initially identified by monoclonal antibodies, has a weight of 45 kDa and is exposed in the membrane of merozoites of *B. bigemina*. GP-45 is a glycosylated protein that is highly immunogenic and therefore more easily recognized by the bovine immune system [8,9]. The sequencing of the gene in a Mexican strain showed that it contains 1058 bp that translate into 351 amino acids [8]. Antibodies from cattle immunized with GP-45 from a Mexican strain and anti-GP-45 monoclonal antibodies bind to merozoites in the homologous strain; however, this binding or recognition does not happen with strains from other countries, which confirms its antigenic polymorphism and indicates a variation of B-cell epitopes among strains [8]. Initially, GP-45 was considered a vaccine candidate, and cattle vaccinated with a purified, native GP-45 generated partial protection, with a lower percentage of parasitemia in cattle that were challenged with the virulent strain compared with unvaccinated cattle [9]. All of this supports the hypothesis that this antigen is involved in the invasion process, despite the presence of mutations in the sequences of GP-45 in some strains around the world [10]. If this antigen is involved in the invasion process, we hypothesize that, as it has a biological function, it contains peptides conserved in field strains from different geographic locations. The use of different bioinformatics tools allows for the generation of essential bioreactives for the knowledge of the biological processes involved in the life cycle of *B. bigemina* and other protozoa [11]. In the present work, we identified GP-45 peptides containing B-cell epitopes conserved in field isolates of *B. bigemina*. Antibodies against these peptides neutralized the invasion process to erythrocytes in vitro. The conserved GP-45 peptides identified here are potential candidates for the development of new diagnostic methods and vaccines.

## 2. Results

### 2.1. Accession Number

The nucleotide sequences obtained from isolates of *B. bigemina* collected in Mexico (Chiapas, Guerrero, Jalisco, Veracruz, and Nayarit), the vaccine strains Seed and Mexico, and Argentina Corrientes isolate were submitted to the GenBank database (National Center for Biotechnology Information, https://www.ncbi.nlm.nih.gov/, accessed on 10 April 2022) with the accession numbers: OM488277, JN049652.1, JN049653.1, JN049650.1, JN049649.1, JN049654.1, JN049655.1, and JN049651.1, respectively.

### 2.2. In Silico Analysis of GP-45 Protein Sequences

The protein sequence of the Seed strain was used as a template to align the sequences corresponding to the rest of the strains and the identity and coverage value (Table 1). The Veracruz (AEJ89907) isolate had the highest identity percentage compared to the vaccinal strain Seed with 99.7%. The Jalisco (AEJ89910) and Mexico (AEJ89911) isolates had an identity percentage of 99.4% when compared to the Seed strain. They were followed by Guerrero (AEJ89909) and Nayarit (AEJ89906) with 99.1%, Mexico JG-29 (AAG28757) with 98.3%, Argentina Corrientes (AEJ89908) with 80% and Chiapas (OM488277) with 94.9% identity compared with the vaccinal strain Seed. The Australia Bond strain (XP_012767625.1) had 95.5% identity compared with the vaccinal strain Seed, but the coverage was 88.6. All the sequences from South Africa are partial sequences, with a total coverage for South Africa Sharpe A17 of 77.5%, while South Africa Khutsong A14, South Africa Devon A04 and South Africa Eikenhof A13 had 78.3% coverage. The similarity percentage for South Africa Eikenhof A13 was 97.1%, for South Africa Devon A04 98.5%, for South Africa Khutsong A14 98.5% and for South Africa Sharpe A17, the similarity percentage was the lowest with 58.9%, compared with the vaccinal strain Seed (Table 1). 

The hydrophobicity analysis of the GP-45 amino acid sequence showed two hydrophobic regions, one positioned at the amino terminal end (amino acids 1 to 17) and the other at the carboxyl terminal (amino acids 338 to 347). The analysis of transmembrane regions with the TMHMM 2.0 program did not show the presence of regions of this type, indicating that the protein is outside the membrane (data not shown). The presence of a signal peptide from amino acids 1 to 22 was identified, which was removed from the sequences at the time of predicting the B-cell epitopes. 

### 2.3. Selection of Predicted Peptides

With the results of the bioinformatics analyses of the GP-45 amino acid sequence, a B-cell epitope prediction was performed. The prediction was performed using the following programs and tools: BcePred, BcePred antigenic tool, ABCPred and the antibody epitope prediction tool of the IEDB. Table 2 shows the sequence of the five peptides predicted by the different bioinformatics programs used, as well as the position in the linear structure in the protein, the number of amino acids, the prediction order and the name assigned. Peptides with order 2 and 3 were the same sequence and number of amino acids, except that an asparagine in peptide 2 was substituted by a lysine in position 57 in peptide 3, and a glutamic acid in position 63 was substituted by a glutamine (Table 2). 

### 2.4. Evaluation of the Specificity of Anti-GP-45 Polyclonal Antibodies by Immunofluorescence

**Figure 1:**
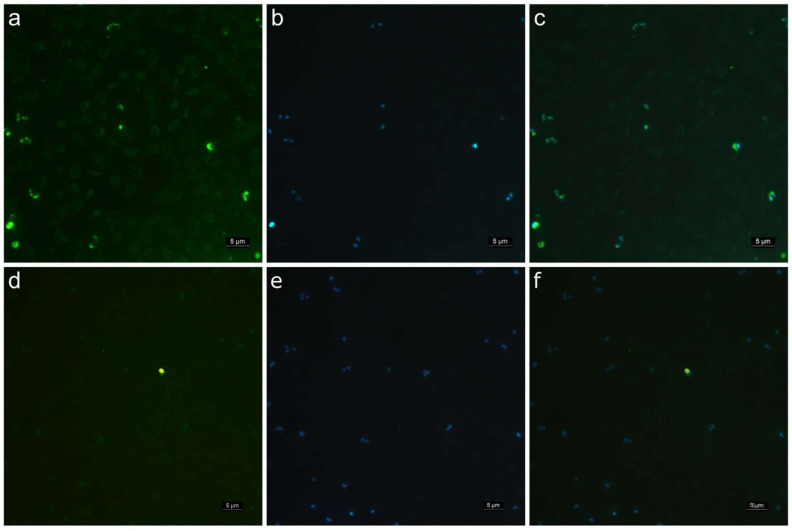
Antibodies anti-GP-45 recognize *B. bigemina* intra-erythrocytic stages. (**a**) Mouse antiserum anti-GP-45. (**c**) Pre-immune mouse serum. Images in panels (**a**,**d**) were taken using a filter for Alexa-488. Images in panels (**b**,**e**) show the same fields as (**a**,**d**), respectively, but with a filter for DAPI. The image in panel (**c**) shows the merge of (**a**,**b**), and the image in panel (**f**) shows the merge of (**d**,**e**). 1000×.

Immunofluorescence slides were performed with *B. bigemina*-infected erythrocytes. Hyperimmune mouse sera recognized a protein present in *B. bigemina* merozoites (Figure 1a). The fluorescence pattern identified merozoites and single-celled trophozoites. The pre-immune mouse sera did not recognize any parasite in the erythrocytes infected with *B. bigemina* (Figure 1c). As a control, DAPI was used to stain the nuclei of the cells (Figure 1b,d).

### 2.5. Evaluation of the Specificity of Anti-GP-45 Polyclonal Antibodies by Western Blot

**Figure 2:**
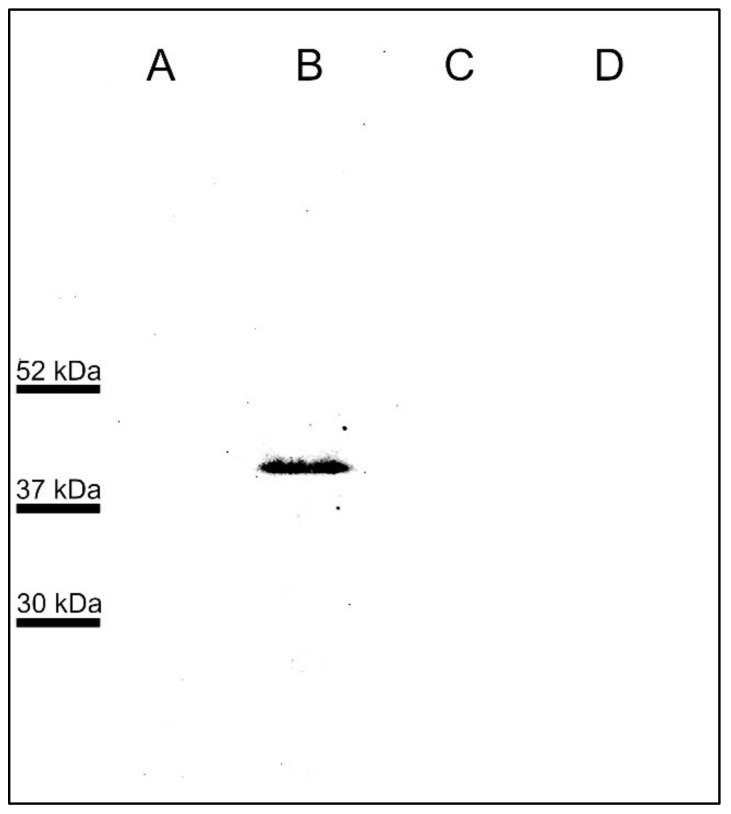
Specificity of anti-GP-45 serum used for expression analysis by Western blot. *B. bigemina*-infected erythrocytes (A,B) and uninfected erythrocytes (C,D) were run on SDS-PAGE and transferred to nitrocellulose membranes. Lanes A and C were incubated with pre-immune serum; lanes B and D were incubated with hyperimmune serum. The molecular weight marker is on the left.

The results from the WB showed a band between 52 kDa and 37 kDa corresponding to the molecular weight of the mature GP-45 protein in an extract of crude antigen from bovine erythrocytes infected with *B. bigemina* and incubated with the hyperimmune serum of a mouse immunized with the GP-45 peptide (Figure 2, lane B). The pre-immune mouse serum did not recognize any protein present in the crude antigen extract of erythrocytes infected with *B. bigemina* (Figure 2, lane A), neither the pre-immune nor the hyperimmune serum recognized any protein in erythrocytes from healthy cattle (Figure 2, lanes C and D, respectively).

### 2.6. Evaluation of the Presence of Anti-Babesia Antibodies in Naturally Infected Cattle

The sera (126) from cattle naturally infected with *B. bigemina* were evaluated by the indirect fluorescence antibody test (IFAT); 81 were positive and 45 were negative from different states of the Mexican Republic. Indirect ELISA using each peptide as an antigen (Table 3) was additionally tested with each serum sample. The GP-45 peptide 1 was recognized by 77/81 positive sera, while 45/45 negative sera did not recognize the peptides. The GP-45 peptide 2 was recognized by 71/81 positive sera, while 45/45 negative sera did not recognize the peptide. The GP-45 peptide 3 was recognized by 66/81 positive sera and by 3/45 negative sera. GP-45-4 was recognized by 66/81 positive sera and by 4/45 negative sera. GP-45-5 was recognized by 73/81 positive sera and by 15/45 negative sera. Peptide 1 showed the highest concordance of 95.06% compared to the gold standard (IFAT) (Table 4). Peptide 5 reached the lowest concordance (81.75%) of the five peptides evaluated. 

The concordance of peptides 1, 2, 3, 4 and 5 compared to the gold standard (IFAT) were 96.83%, 92.06%, 85.71%, 84.92% and 81.75%, respectively, with peptide 1 reaching the highest concordance and peptide 5 with the lowest percentage of agreement. The peptide with the highest positive predictive value (PPV) was peptide 1 and the lowest PPV was peptide 5. The peptides with the lowest negative predictive value (NPV), peptides 3 and 4, obtained NPVs of 73.68% and 71.21%, respectively.

### 2.7. Evaluation of the Neutralizing Ability of Anti-Babesia Antibodies In Vitro Culture of Erythrocytes Infected with B. bigemina

**Figure 3:**
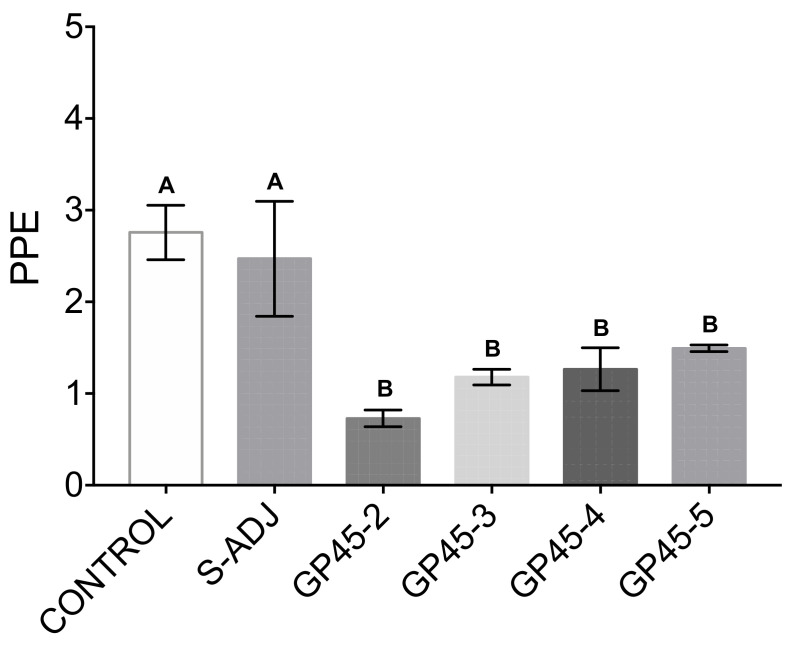
Sera from Gp-45-immunized rabbits reduce erythrocyte invasion by *B. bigemina* in vitro. Control group received added culture medium; S-ADJ group was supplemented with serum from a rabbit immunized with adjuvant; GP-45-2 group was supplemented with the serum of a rabbit immunized with peptide GP-45-2; GP-45-3 group was supplemented with serum of a rabbit immunized with the peptide GP-45-3; GP-45-4 group was supplemented with serum of a rabbit immunized with the peptide GP-45-4 and GP-45-5 group was supplemented with serum of a rabbit immunized with the peptide GP-45-5. Statistical analysis was performed with an ANOVA with Tukey’s test with a 95% confidence. Equal letters do not have statistical differences, different letters indicate statistical difference.

The antisera from rabbits immunized with the different GP-45 peptides of *B. bigemina* were evaluated for their ability to neutralize a parasite invasion to erythrocytes in vitro. All four peptides evaluated generated antibodies that decreased the percentage of infected erythrocytes compared to the control groups (Figure 3). In the control group, a 2.757 percentage of parasitized erythrocytes (PPE) was achieved. This group was not supplemented with the rabbit serum. The S-ADJ group was supplemented with the serum from a rabbit immunized only with adjuvant and was used as a control for the rabbit serum effect in the culture medium. It reached 2.471 PPE, being statistically the same as the control group without the rabbit serum. The groups supplemented with the hyperimmune sera of rabbits immunized with adjuvant and the peptides GP-45 2, GP-45 3, GP-45 4 and GP-45 5, were statistically different from the control group, and S-ADJ reaching 0.729, 1.180, 1.265 and 1.494 of PPE, respectively, the GP-45 2 group being the one that managed to reduce the PPE to the lowest, with no statistical differences among them (Figure 3). An ANOVA and Tukey’s test were performed with a 95% confidence interval.

## 3. Discussion

Bovine babesiosis is a tick-borne disease caused in the Americas by *B. bovis* and *B. bigemina* [12]. To date, few studies have been carried out on the genes encoding *B. bigemina* proteins involved in a merozoite invasion to erythrocytes. For this reason, this work aimed to identify B-cell epitopes in the polymorphic GP-45, which were conserved in different isolates from Mexico and other countries. The fourteen protein sequences analyzed in this work correspond to the sequences obtained in this study and those previously reported and deposited in the databases. The result of the multiple alignment analysis allowed us to identify regions of the protein that were conserved in all isolates and strains. A signal peptide prediction analysis showed a sequence cleavage site corresponding to the signal peptide, as previously reported [8]. Hydrophobicity analyses allowed us to identify the exposed regions of the protein and, therefore, the hydrophilic regions of the protein exposed to antibodies [13]. The results of the immuno-bioinformatic analyses allowed for the identification of five peptides that were immunogenic and predicted as B-cell epitopes. These peptides were located in the extracellular region of the protein, which was identified by transmembrane domain prediction analysis, a tool that has previously been used and validated [14]. Sera from mice immunized with a GP-45 peptide specifically recognized *B. bigemina* merozoites. So far, there is no report of the pattern of expression of GP-45 in *B. bigemina*. However, GP-45 is known to be a membrane protein, and this coincides with the stain pattern observed in the immunofluorescence assay and with the fact that proteins with membrane anchor sites by GPI are expressed in the membrane and apical complex of merozoites [8,15,16,17]. The specificity of this antibody was confirmed by Western blot where the serum of mice immunized with the same GP-45 peptide identified a band between 37 and 50 kDa in protein extracts of erythrocytes infected with *B. bigemina*. These results agree with the expected molecular weight of GP-45, which is 45 kDa and with those observed in different studies carried out with GP-45 in *B. bigemina*, where a single band below 50 kDa was identified [8,9,18]. However, our results do not agree with reports where, by Western blot, several bands have been identified when analyzing membrane-anchored proteins because they are processed [16,17]. This may be because we used a polyclonal serum against a single peptide, which does not identify the processed protein. Perhaps by using antibodies against other regions of the protein, it can be verified if there is processing of GP-45.

The results obtained with the indirect ELISA test for the detection of antibodies against *B. bigemina* are similar to the tests currently published for the detection of antibodies against other protozoa such as *Toxoplasma gondii* [19]. The ELISA test developed in the present work reached 100% diagnostic specificity using synthetic peptides belonging to a hydrophilic region of the GP-45 protein of *B. bigemina*. The indirect ELISA test based on the peptide one developed in the present work for the diagnosis of *B. bigemina* reached sensitivity values higher than those obtained by the competitive (commercial) ELISA test based on an epitope of RAP-1 from *B. bovis*, which achieves 60% sensitivity using bovine sera from different parts of Mexico [20]. The indirect ELISA test based on this peptide has 35% more sensitivity to detect *B. bigemina* than the competitive (commercial) ELISA test based on an epitope of RAP-1 for the detection of *B. bovis*. However, the indirect ELISA test based on the recombinant SBP-4 protein for the detection of *B. bovis* reached 98.7% of diagnostic sensitivity using sera from different parts of Mexico [20]. Therefore, the diagnostic sensitivity of that test for that species exceeded the values obtained in the present work for the detection of antibodies against *B. bigemina* using the indirect ELISA based on the GP-45 peptide one of *B. bigemina*. Castillo-Pérez et al. [21] developed the indirect ELISA test using the recombinant protein RAP-1a of *B. bigemina* for the detection of antibodies against this parasite reaching 90% sensitivity and 98.75% specificity. The indirect ELISA test evaluated by Kim et al. [22] using the recombinant rMSA-2c *B. bovis* and rRAP-1/CT17 from *B. bigemina* reached 96.7% sensitivity and 95% and 93.8% specificity, respectively. The values obtained in this work are similar in sensitivity, however, the specificity was exceeded by 6.2% [22]. All the evaluated peptides have a concordance greater than 70%, which suggests that any of the five can be used for the detection of antibodies against *B. bigemina* according to the OIE [23] guidelines for the development of diagnostic tests for transmitted diseases by vectors such as bovine babesiosis.

In *B. bovis*, it has been shown that antibodies against the GP-anchored proteins of merozoites can generate antibodies that neutralize the merozoite invasion of erythrocytes [24,25]. The results obtained in this work identified the presence of B-cell epitopes in *B. bigemina* GP-45. We evaluated the neutralizing capacity of polyclonal antibodies against four peptides containing predicted B-cell epitopes. All four peptides induced antibodies that neutralized a merozoite invasion to erythrocytes. Peptide 2 and peptide 3 are sequences conserved in different isolates, which were different by one amino acid. Importantly, antibodies against both peptides induced neutralization, which means that despite their difference in sequence, they were still able to induce antibodies that neutralize invasion. Peptide four and peptide five are the most conserved peptides, and except for the sequences from South Africa, they are fully conserved in all isolates and strains. The sequences reported from South Africa are short fragments and were obtained by nested PCR and direct PCR sequencing, which introduces mutations, and are not generally accepted to report sequences, which is normally done by cloning. We did not evaluate antibodies against peptide 1 since it was not present in all the sequences analyzed because some reported sequences were incomplete, and this peptide was located at the carboxyl terminus (amino acids: 283–301). All four peptides induced antibodies with neutralization capacity when we use an in vitro cultured strain from Argentina to perform the neutralization assays. All this is consistent with ideal characteristics of vaccine candidates against the disease [26]. Still, the variability in the amino acid sequence of GP-45, and the fact that some strains and isolates of *B. bigemina* do not express GP-45 or do not contain the gene, has discouraged its inclusion as a vaccine candidate or diagnostics tool [8,10]. Taken together, the presence of B-cell epitopes in strains from different parts of the world, the capacity of GP-45 to induce neutralization-sensitive antibodies and the presence of field isolates and laboratory strains that express GP-45 need further investigation.

## 4. Materials and Methods

### 4.1. Evaluation of the Presence of Anti-Babesia Antibodies in Naturally Infected Cattle

Eight different *B. bigemina* isolates and strains were processed in this work: Six isolates, of which five are from states in Mexico, were used: Santiago Ixcuintla (Nayarit); Tapalpa (Jalisco); Acayucan (Veracruz); Pungarabato (Guerrero); Cintalapa (Chiapas). These isolates were obtained from infected blood or ticks. The isolate from Argentina was obtained from ticks from the city of Chavarria, province of Corrientes, Argentina. Two strains were also included: a virulent strain maintained in the laboratory called Kutler, collected in Mexico, and the vaccine strain Seed, an attenuated strain, which is maintained in Mexico in in vitro culture. The *B. bigemina* DNA samples were obtained through a mixture of phenol–chloroform–isoamyl alcohol and precipitated by adding cold absolute ethanol and incubated at −20 °C overnight. The supernatant was discarded, and the pellet was washed with ethanol: cold water (70:30 *v:v*) and resuspended in 50 µL of nuclease-free water. The primers used for the amplification of *B. bigemina GP-45* were designed to align in a specific region of the locus outside the ORF of the gene. The sequence of the primer forward (GP-45-F) is 5’-GCACCTGCAAAGCAATGTAGC-3’ and the sequence of the primer reverse (GP-45-R) is 5’-CGCAGTTGATCGCGTGTC-3’. The PCR was performed with 12.5 µL of Master Mix 1X (50 units/mL of DNA polymerase (pH 8.5), 400 µm of: dATP, dGTP, dCTP, dTTP and 3 mM MgCl_2_), 1 µL of each primer (GP-45-F and GP-45-R) at 10 µM/mL and 1 µL of template DNA at <250 ng/mL and nuclease-free water was added until reaching a final volume of 25 µL. The thermocycling process for gene amplification was performed under the following conditions: 5 min of an initial denaturation at 95 °C, followed by 35 cycles of amplification; 30 s at 94 °C for denaturation, followed by 30 s at 60 °C for alignment and 30 s at 72 °C for extension. At the end of the cycles, a final extension for 10 min at 72 °C was added. The PCR products were run on a 1.5% agarose gel stained with ethidium bromide and visualized under UV light. Cloning of the gene was performed with the TOPO TA cloning kit (plasmid CR^®^2.1-TOPO and *E. coli* strain TOP10) (Invitrogen, Carlsbad, CA, USA). Ten white colonies were selected and used to isolate and purify the plasmids containing the insert. The cloning products were sequenced in both directions using each of the primers by the automated Sanger method. The electropherograms were analyzed and edited with the Chromas lite (Technelysium Pty Ltd., South Brisbane, Queensland, Australia), Vector NTI Advance TM 10 (Invitrogen, Carlsbad, CA, USA) and CLC Main Workbench 6.8 (QIAGEN Bioinformatics, Aarhus, Denmark) program. The nucleotide sequences were analyzed to localize the open reading frames (ORF) using the ORF finder (National Center for Biotechnology Information) (https://www.ncbi.nlm.nih.gov/orffinder/ accessed on 10 April 2022) [27] and they were translated into amino acid sequences.

### 4.2. In Silico Analysis of Predicted GP-45 Protein Sequences

Fourteen amino acid sequences of GP-45 were used; eight were obtained in this work and six were obtained from the Protein Database website: XP_012767625.1 (Australia), AGU67944, AGU67947, AGU67946, AGU67945 (South Africa), AAG28757 (Mexico). The degree of conservation of the proteins was performed by a multiple sequence alignment using Clustal Omega (https://www.ebi.ac.uk/Tools/msa/clustalo/, accessed on 10 April 2022) [28]. Hydrophilic and hydrophobic regions were predicted using the TMpred program (https://bio.tools/TMPred/, accessed on 10 April 2022) [29] and Protscale (https://web.expasy.org/protscale//, accessed on 10 April 2022) using the Kyte and Doolittle algorithm [13,30]. Transmembrane regions were predicted using the TMHMM tool (https://services.healthtech.dtu.dk/service.php?TMHMM-2.0/, accessed on 10 April 2022) [31]. The prediction of secondary structures was performed through the NNpredict program (http://130.88.97.239/bioactivity/nnpredictfrm.html/, accessed on 10 April 2022); the myristylation, glycosylation and phosphorylation sites with the PROSITE program (https://prosite.expasy.org, accessed on 10 April 2022) [32]. The presence of a signal peptide was predicted by using the SignalP 4.1 Server tool (https://services.healthtech.dtu.dk/service.php?SignalP-4.1/, accessed on 10 April 2022) [33,34]. Functional domains were searched using the Pfam tool (https://pfam.xfam.org, accessed on 10 April 2022) [35,36] and SMART (http://smart.embl-heidelberg.de, accessed on 10 April 2022) [37,38]. The prediction of conserved B-cell epitopes was performed with different bioinformatic algorithms. Hydrophobicity was determined using the Parker method of the BcePred program (http://crdd.osdd.net/raghava/bcepred/, accessed on 10 April 2022) [39,40] and was determined using the Kolaskar method algorithm of the BcePred program and the antigenic tool (http://www.bioinformatics.nl/cgi-bin/emboss/antigenic, accessed on 10 April 2022) [39,41]. The prediction of linear B-cell epitopes was performed using both the ABCPred program (http://crdd.osdd.net/raghava/abcpred/, accessed on 10 April 2022) [42] and the antibody epitope prediction tool of the IEDB server (http://tools.immuneepitope.org/bcell/, accessed on 10 April 2022) [43]. A BLAST analysis was performed on the NCBI portal (https://blast.ncbi.nlm.nih.gov/Blast.cgi, accessed on 10 April 2022) and a BLAST on the Sanger Institute portal (https://www.sanger.ac.uk/action/BLAST, accessed on 10 April 2022) [44]. The sequence match search was performed with the BLAST tool (Basic Local Alignment Search Tool) in the sequences available in the NCBI database. Only the peptides with the best scores in the bioinformatic analyses and that were found specifically in *B. bigemina* GP-45 were selected and evaluated.

### 4.3. Generation of Hyperimmune Sera against GP-45 Peptides in Rabbits and Mice

This study was approved by the Institutional Subcommittee for the Care of Animals in Experimentation under the Mastery and Doctorate program in Sciences of Production and Animal Health of the National Autonomous University of Mexico (document: MC/2014-38), and by the Bioethics Committee of the Natural Sciences College of the Autonomous University of Queretaro (121FCN2018). Each of the peptides containing the conserved B-cell epitopes were generated by chemical synthesis in the Multi-Antigenic Peptide system with eight branches (MAP-8) (GL Biochem Ltd., Shanghai, China). Two-month-old New Zealand rabbits were used. Each rabbit was immunized with 100 µg of peptide in 500 µL of 1x PBS (13.6 mM NaCl, 2.6 mM KCl, 8.33 mM NaH_2_PO_4_ pH 7.2) and 500 µL Montanide ISA 71 adjuvant VG (Seppic, Paris, France). Two rabbits were immunized with each peptide subcutaneously. Four immunizations were performed every 15 days. Ten days after the last immunization, blood samples were taken to obtain rabbit hyperimmune sera. Mice were immunized with the same peptides as the rabbits. Five mice were immunized subcutaneously with 100 µg of each peptide in 50 µL of 1X PBS and 50 µL Montanide ISA 51 adjuvant VG. Four immunizations were performed every 21 days. Hyperimmune sera were collected 10 days after the last immunization.

### 4.4. Evaluation of the Specificity of the Antibodies Present in Mouse Polyclonal Sera

The specificity of the antibodies in the sera of the immunized rabbits was evaluated by indirect immunofluorescence (IIF) and immunoblotting (WB). To obtain the *B. bigemina* antigen for IIF and WB, a splenectomized calf was experimentally infected with the Las Torres, Michoacan isolate of *B. bigemina* [45]. When the parasitemia reached 20%, the jugular vein was punctured with a needle and was collected in a sterile flask. To defibrinate the blood, glass beads were added, and the flask was gently shaken. Erythrocytes were washed with VYM solution [46] and kept at −20 °C or they were used to prepare blood smears using glass slides, as reported previously [47]. To determine whether antibodies from mice immunized with GP-45 peptides recognize the native protein, an indirect immunofluorescence analysis was carried out. Blood smears were permeabilized with 100% acetone for 1 h at −20 °C. Pre-immune and hyperimmune sera diluted 1: 200 in PBS-T were incubated for 1 h at 37 °C. Anti-mouse IgG antibodies coupled to Alexa Fluor 488 were diluted 1:200 with DAPI at 1 ug/mL and incubated for 1 h at 37 °C. Three washes were carried out for 5 min in PBS-T with shaking. The mounting of the slides was carried out with phosphated glycerin 1: 1 (glycerin: PBS 2×). The slides were observed at 100× in a LEICA microscope, DM 2500 and LEICA DFC420 camera (Leica Microsystems, Heerbrugg, Switzerland) with filters for Alexa Fluor 488 and DAPI. The images were acquired and processed with LEICA Application Suite LAS V4.2 software.

### 4.5. Specificity of Anti-GP-45 Mouse Sera Used for Expression Analysis

To confirm the expression of GP-45 in *B. bigemina* merozoites, a WB analysis was performed using the protocol published by Hidalgo Ruiz et al. [48]. *B. bigemina*-infected erythrocytes were lysed by freeze–thawing cycles followed by centrifugation in PBS. Protein samples were mixed with 2× Laemmli buffer and boiled for 10 min. SDS-PAGE electrophoresis gel 12% (29% acrylamide: 1% bisacrylamide) was performed using running buffer (3.5 mM SDS, 200 mM Glycine, 25 mM Tris Base, pH 8.3). The Precision Plus Protein™ Dual Color Standards (Bio Rad, Hercules, CA, USA) was used as a molecular weight marker. Separated proteins from the SDS-PAGE were transferred to a nitrocellulose membrane (Bio Rad, Hercules, CA, USA) at 20 volts for 20 min using a transfer buffer (25 mM Tris base, glycine 200 mM, 25 mM Tris base, methanol 20%, pH 8.3) in a Trans-Blot^®^ SD Semi-Dry Electrophoretic Transfer Cell (Bio Rad, Hercules, CA, USA). Transfer was verified with Ponceau Red stain (Ponceau red 0.2 % *w/v* acetic acid 5% *v/v*). The membrane was washed three times for 5 min at 250 rpm at 4 °C with TBS-T 1X (150 mM NaCl, 10 mM Tris Base, 0.1 % *v/v* Tween 20, pH 7.5). The membrane was blocked with blocking buffer (TBS-T 0.05% with 5% skim milk) and shaking at 250 rpm overnight at 4 °C. Next, the membrane was washed 5 times for 10 min with TBST 0.1% at 4 °C for 10 min with shaking at 250 rpm. The membrane was incubated with pre-immune or post-immune mouse sera at 1:5000 in TBS-T 1 h at 37 °C with shaking. The washing process was repeated, and the membrane was incubated with the secondary antibody. The plates were incubated with an anti-mouse IgG (H + L) antibody coupled with peroxidase (Jackson ImmunoResearch, Baltimore, MD, USA) for 1 h at 37 °C, diluted 1:10000 in TBS-T under the same incubation conditions as the primary antibody. The washing process was repeated. The immunodetection was performed by the Amersham ECL Western Blotting Detection Reagent (Cytiva, Piscataway, NJ, USA). The images were acquired with a ChemiDoc MP Imaging System (Bio Rad, Hercules, CA, USA).

### 4.6. Recognition of GP-45 Peptides by Antibodies Present in Cattle Naturally Infected with B. bigemina

To identify cattle naturally infected with *B. bigemina*, the following protocol was performed: first, serum was collected from cattle living in endemic areas of bovine babesiosis. The sera were collected in four states in Mexico: Aguascalientes, Queretaro, Sinaloa, and Veracruz. Sera were evaluated by the IFAT according to the published protocol [23] with a dilution of the sera of 1:80. They were subsequently incubated with the goat anti-IgG secondary antibody conjugated with Alexa Fluor-488 (Jackson ImmunoResearch, Baltimore, MD, USA) 1:100 dilution for one hour at 37 °C. Only sera positive for *B. bigemina* antibodies and negative for B. bovis antibodies were used in the experiment. The recognition of GP-45 peptides by bovine sera was determined by indirect ELISA using the protocol published by Mosqueda et al. (2019). GP-45 peptides were bound in a Corning 3590 Polystyrene Flat Bottom 96-Well High Bind EIA/RIA Clear Microplate (Corning Inc., NY, USA) at a concentration of 10 µg/mL in carbonate/bicarbonate buffer (0.1 M sodium carbonate, 0.1 M sodium bicarbonate) and incubated overnight at 4 °C. The plates were washed with PBS-T 0.05% 3 times for 30 s and dried on an absorbent surface. The plates were blocked with 5% (*w/v*) skim milk in PBS-T for 1 h at 37° C with shaking at 250 rpm. The washing process was repeated. Each cattle serum was diluted 1:40 in PBS-T 0.05% and incubated for 1 h at 37 °C with shaking at 250 rpm. After washing, the plates were incubated with anti-bovine IgG (H + L) antibody coupled with peroxidase (Jackson ImmunoResearch, Baltimore, MD, USA) for 1 h at 37 °C with shaking at 250 RPM, and at 1:1000 dilution in PBS. The washing process was repeated. The signal was detected by adding 0.4 mg/mL OPD (Sigma-Aldrich, St. Louis, MO, USA) as substrate. The plates were read on an ELISA Microplate Reader (Bio Rad, Hercules, CA, USA) at 450 nm, 20 min after the development solution was added. Each serum was analyzed in triplicate. The cut-off points were calculated using the negative reference control mean values. All sera with absorbance above the cut-off point were considered positive and all sera with absorbance below the cut-off point were considered negative for the presence of antibodies against *B. bigemina* GP-45. The formula used to calculate the cut-off point is: mean + 3 standard deviations from a negative reference serum. The cut-off point was calculated for each peptide on each plate. A 2 × 2 contingency table was made to compare the results obtained by the ELISA test against the IFAT results using the formulas to determine sensitivity, specificity and concordance [49]. The presence of antibodies against GP-45 peptides was determined in bovines naturally infected with *B. bigemina*. The true positive, false positive, false negative, true negative, sensibility, specificity, concordance, positive predictive value and negative predictive value were calculated with the formulas shown in Table 4.

### 4.7. In Vitro Neutralization Assay

To analyze whether antibodies against conserved peptides of GP-45 can block the invasion of Babesia merozoites, an in vitro neutralization assay was performed. A culture of the Argentina strain of *B. bigemina* was used [50]. The neutralization test was modified as follows: The merozoites were grown in 96-well culture plates with 297 µL of HL-1 medium (5% bovine erythrocytes, 40% bovine serum, 0.1 M of TAMPO and pH 7.2) and 22.5 µL of anti-GP-45 serum. The cultures were incubated at 37 °C with 5% CO_2_. When parasitemia reached 6%, 16.5 μL of medium with erythrocytes and decomplemented rabbit serum were added. Each culture was performed in triplicate. The medium was changed daily by removing 180 µL and adding 171 µL of fresh culture medium and 9 µL of hyperimmune rabbit sera per well. The cultures were incubated for 72 h at 37 °C with 5% CO_2_. After 72 h of incubation time, smears of the culture were made, fixed with absolute methanol and stained with Giemsa stain. Two thousand erythrocytes were counted from each well for each treatment. An ANOVA was performed with the Tukey’s test to determine the statistical differences of the cultures incubated with hyperimmune rabbit sera to each of the peptides compared to the control. The data were analyzed using Prism GraphPad 9 Software.

## 5. Conclusions

GP-45 contains peptides with conserved B-cell epitopes in isolates and strains of *B. bigemina* from different geographic origins including Mexico, Argentina and Australia. GP-45 generates specific antibodies that recognize proteins in erythrocytes infected with *B. bigemina* by indirect immunofluorescence and a Western blot. Sera from cattle naturally infected with *B. bigemina* contain antibodies that bind to specific peptides of GP-45. Peptide-specific antisera to GP-45 neutralize a merozoite invasion of erythrocytes in vitro.

## Figures and Tables

**Table 1:** Percentage of identity and total coverage between predicted GP-45 protein sequences in different *B. bigemina* strains from Mexico, Argentina, Australia and South Africa compared to the Seed strain.

Isolate/Strain		Seed	
	GenBank ID	Coverage	Identity
Veracruz	AEJ89907	100%	99.70%
Jalisco	AEJ89910	100%	99.40%
Mexico	AEJ89911	100%	99.40%
Guerrero	AEJ89909	100%	99.10%
Nayarit	AEJ89906	100%	99.10%
Mexico JG-29	AAG28757	100%	98.30%
Argentina Corrientes	AEJ89908	100%	98.00%
Chiapas	OM488277	100%	94.90%
Australia Bond	XP_012767625.1	88.60%	95.50%
South Africa Khutsong A14	AGU67945	78.30%	98.50%
South Africa Devon A04	AGU67946	78.30%	98.50%
South Africa Eikenhof A13	AGU67947	78.30%	97.10%
South Africa Sharpe A17	AGU67944	77.50%	58.90%

**Table 2:** Predicted peptides selected by bioinformatics.

Prediction Order	Position (aa)	Length (aa)	Amino Acid Sequence
1	282–301	19	ERAVSGATTHGGDARGVNP
2	52–70	18	MHIVSNLLDVEPIVGMYG
3	52–70	18	MHIVSKLLDVQPIVGMYG
4	142–159	17	GFLSTATDVPESDLAKK
5	178–195	17	NLQMFLKVFYNKNSPLF

**Table 3:** Evaluation of GP-45 peptides by indirect ELISA by *B. bigemina* positive and negative sera. Presence of antibodies against each peptide naturally infected bovine sera. “+” positive; “−“ negative.

*n* = 126	Estate	Ranch/Farm	Total	Peptide 1		Peptide 2		Peptide 3		Peptide 4		Peptide 5		Total
				“+”	“−”	“+”	“−”	“+”	“−”	“+”	“−”	“+”	“−”	
**IFAT Positive *n* = 81**	**Aguascalientes**	**Villa Guadalupe**	35	7	0	7	0	5	2	6	1	7	0	7
		Las Palomas		23	1	20	4	17	7	14	10	19	5	24
		Granja María I		4	0	3	1	2	2	3	1	3	1	4
	Querétaro	Granja Areceli	2	2	0	2	0	2	0	2	0	2	0	2
	Sinaloa	El Torito	20	6	2	7	1	6	2	7	1	7	1	8
		La Herradura		2	0	2	0	2	0	2	0	2	0	2
		El Barón		2	0	2	0	2	0	2	0	2	0	2
		El Moral		8	0	8	0	8	0	7	1	8	0	8
	Veracruz	El Arbolito	24	2	0	2	0	2	0	2	0	2	0	2
		Playa Vicente		2	0	2	0	1	1	2	0	2	0	2
		La Esperanza		2	1	2	1	3	0	2	1	2	1	3
		Las Torres		1	0	1	0	1	0	1	0	1	0	1
		San Faudila		3	0	2	1	3	0	3	0	3	0	3
		El orijuelo		11	0	9	2	10	1	11	0	11	0	11
		Buenos Aires		2	0	2	0	2	0	2	0	2	0	2
	Total Positive/Negative		81	77	4	71	10	66	15	66	15	73	8	81
IFAT Negative *n* = 45	Querétaro	Amazcala	16	0	6	0	6	0	6	0	6	0	6	6
		Los Moreno		0	10	0	10	0	10	0	10	0	10	10
	Durango	Arroyo Seco	29	0	29	0	29	3	26	4	25	15	14	29
	Total Positive/Negative		45	0	45	0	45	3	42	4	41	15	30	45

**Table 4:** Concordance and contingency table. TP: True Positive sera; FP: False Positive sera; FN: False Negative sera; TN: True Negative sera; PPV: Positive Predictive Value; NPV: Negative Predictive Value.

Parameter	Formula	Peptide 1	Peptide 2	Peptide 3	Peptide 4	Peptide 5
TP	a	77	71	66	66	73
FP	b	0	0	3	4	15
FN	c	4	10	15	15	8
TN	d	45	45	42	41	30
% Sensitivity	a/(a + c) * 100	95.06	87.65	81.48	81.48	90.12
% Specificity	d/(b + d) * 100	100.00	100.00	93.33	91.11	66.67
% Concordance	(a + d)/(a + b + c + d) * 100	96.83	92.06	85.71	84.92	81.75
PPV	a/(a + b) * 100	100.00	100.00	95.65	94.29	82.95
NPV	d/(c + d) * 100	91.84	81.82	73.68	73.21	78.95
Total = *n*	a + b + c + d	126	126	126	126	126

## Data Availability

The nucleotide sequence data reported in this article were submitted to the GenBank database under the accession numbers.
